# Deep reinforcement learning for data-driven adaptive scanning in ptychography

**DOI:** 10.1038/s41598-023-35740-1

**Published:** 2023-05-30

**Authors:** Marcel Schloz, Johannes Müller, Thomas C. Pekin, Wouter Van den Broek, Jacob Madsen, Toma Susi, Christoph T. Koch

**Affiliations:** 1 Institute of Physics and IRIS Adlershof, Humboldt Universität zu Berlin, Newtonstraße 15, 12489 Berlin, Germany; 2 Faculty of Physics, University of Vienna, Boltzmanngasse 5, 1090 Vienna, Austria

**Keywords:** Characterization and analytical techniques, Software, Transmission electron microscopy

## Abstract

We present a method that lowers the dose required for an electron ptychographic reconstruction by adaptively scanning the specimen, thereby providing the required spatial information redundancy in the regions of highest importance. The proposed method is built upon a deep learning model that is trained by reinforcement learning, using prior knowledge of the specimen structure from training data sets. We show that using adaptive scanning for electron ptychography outperforms alternative low-dose ptychography experiments in terms of reconstruction resolution and quality.

## Introduction

Ptychography is a coherent diffractive imaging (CDI) method that has found use in light, X-ray and scanning transmission electron microscopies (STEM). The method combines diffraction patterns from spatially overlapping regions to reconstruct the structure of a specimen for arbitrarily large fields of view^1^, with many advantages over other imaging methods^2–5^. The development of new hardware^6,7^ and reconstruction algorithms^8,9^ has led to ptychography becoming a mature electron microscopy technique^4^. Current research to further improve it is driven by the desire to investigate thick samples^10–14^ as well as to lower the required electron dose^15–18^. In order to lower the dose, researchers have tried to vary various experimental parameters while preserving information redundancy through overlapping probes. One approach involves a defocused probe rastered across the specimen with a less dense scan pattern. This uses a lower dose than focused probe ptychography, but introduces additional complications for the reconstruction algorithm due to an increased need to account for partial spatial coherence in the illuminating probe^18^. Another approach is simply to scan faster by lowering the dwell time per probe position, an overall decrease in dose can be realized. However, this comes with its own challenges, as the physical limits of the electron source, microscope, and camera all must be considered. Finally, a third approach is the optimization of the scan pattern, deviating from a raster grid in favour of a generally more efficient pattern^19^. This approach can, however, only yield a limited improvement in reconstruction quality as it is not capable of taking into account the structure of the specimen in the scan pattern.

**Figure 1:**
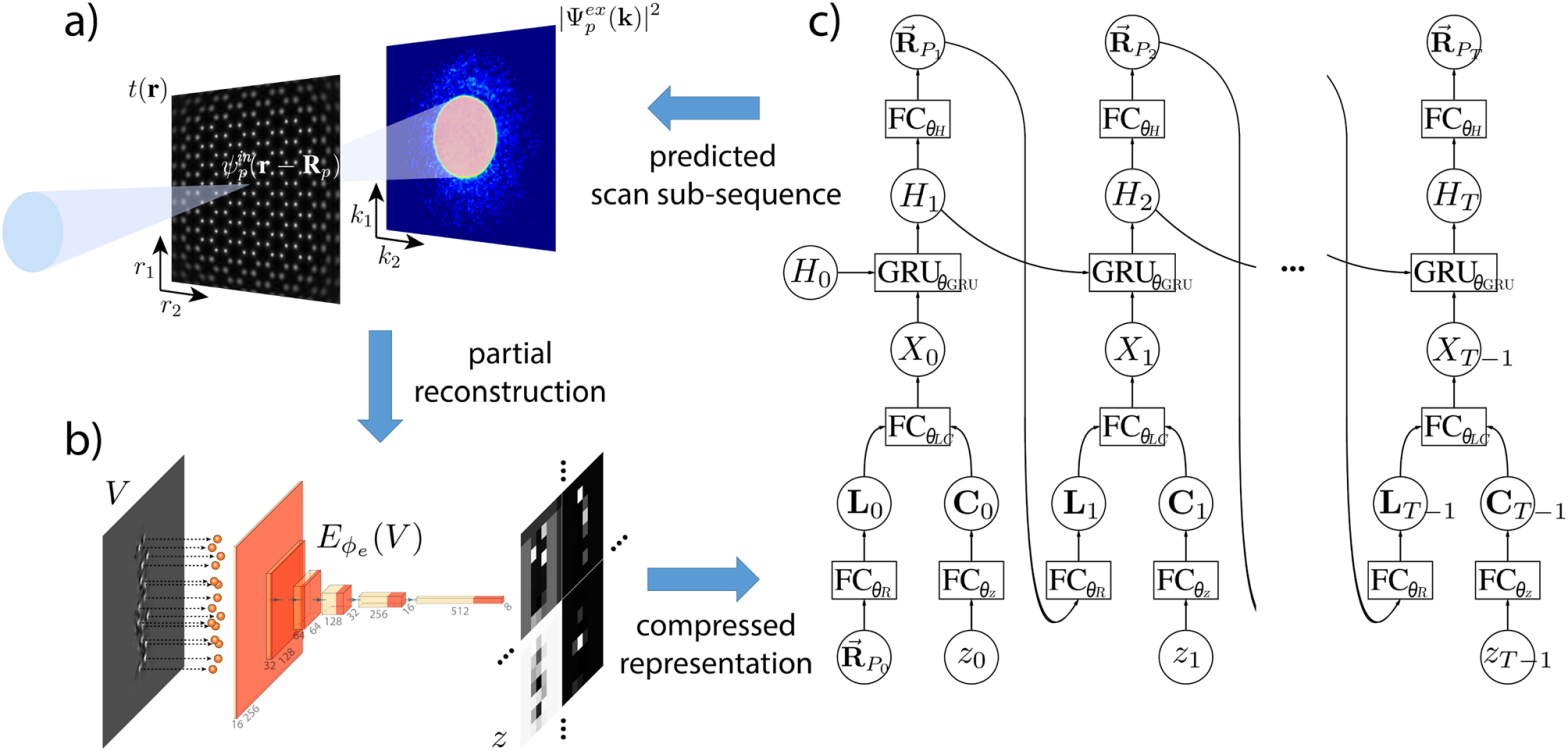
Schematic of the adaptive scanning workflow with its three main components. (**a**) Experimental data acquisition in ptychography. At the scan position ${\textbf {R}}_p$ of the scan sub-sequence $\vec {{\textbf {R}}}_{P_t}$, the beam illuminates a sample, where the incident electron wave $\psi ^{in}_p({\textbf {r}}-{\textbf {R}}_p)$ interacts with the transmission function $t({\textbf {r}})$. The wave exiting the sample is propagated by a Fourier transform to the detector located in the far field and the intensity $I_p = |\Psi ^{ex}_p({\textbf {k}})|^2$ is recorded as a diffraction pattern. (**b**) A reconstruction *V* generated from diffraction patterns of a scan sub-sequence is mapped to the compressed representation *z* by using an encoder network $E_{\phi _e}(V)$. (**c**) Schematic of the forward propagation process of the RNN model. The RNN consists of GRU units that use the hidden state $H_t$ from the previous time step and the hybrid input information $X_t$ to create a new hidden state $H_{t+1}$. The hybrid input is the concatenation of the pre-processed information from the sub-sequence of scan positions $\vec {{\textbf {R}}}_{P_t}$ and the corresponding compressed representation of the partial reconstruction $z_t$. The output of the GRU cell is used to predict the positions of the next sub-sequence $\vec {{\textbf {R}}}_{P_{t+1}}$ and is also used as the input for the next GRU cell.

In this work we present an approach particularly tailored for electron ptychography that enables reduction of the electron dose through adaptive scanning. It is based upon the idea that, at atomic resolution, ptychography requires an increased information redundancy through overlapping illuminating beams only in regions that contain the atomic structure of the scanned specimen. We present here a workflow that scans only the regions with the highest information content in order to strongly improve the ptychographic reconstruction quality while keeping low the total number of scan positions, and therefore the total dose. The scan positions are predicted sequentially during the experiment and the only information required for the prediction process is the diffraction data acquired at previous scan positions. The scan position prediction model of the workflow is a mixture of deep learning models, and the model training is performed with both supervised and reinforcement learning (RL). A schematic of the workflow is given in Fig. 1. The synergy of deep learning and RL has already shown strong performance in various dynamic decision-making problems, such as tasks in robotics^20,21^ or visual recognition^22–24^. The success of this approach, despite the complexity of the problems to overcome, can be attributed to the algorithms’ ability of learning independently from data. Similarly, the proposed algorithm here solves a sequential decision-making problem by learning from a large amount of simulated or, if available, experimental ptychographic data consisting of hundreds to thousands of diffraction patterns. Here, the focus of the learning is specifically designed to maximize the dynamic range in the reconstruction for each individual scan position. The algorithm then transfers the learned behaviour it developed offline to a realistic experimental environment.

Our approach is conceptually related to the subfield of computer vision that focuses on identifying relevant regions of images or video sequences for the purpose of classification or recognition. However, there are fundamental differences not only in the purpose, but also in the solution strategy for our application in contrast to computer vision tasks. Differences include a lack of direct access to images (updated real space information is only accessible through a highly optimized reconstruction algorithm), non-optimal parameter settings of the reconstruction algorithm and experimental uncertainties such as imprecise scan positioning of the microscope or contamination of the specimen requiring pre-processing of the reconstructed image, and the necessity of a much larger number of measurements requiring methods that improve the performance of the sequential decision making process. Work in adaptive scanning for X-ray fluorescence imaging^25^ and for scanning probe microscopy^26^ has also recently been reported. However, the work in Ref.^25^ is more closely related to previous work in scanning electron microscopy that divides the measurement into a low-dose raster scan and a subsequent high-dose adaptive scan^27^. For the latter work in Ref.^26^, it has been reported that its model suffers in performance as it lacks prior knowledge of the domain structure, which can be compensated by including a deep learning model with domain specific knowledge. Our proposed approach is the first application of adaptive scanning to ptychography, and is further unique in that the scan pattern is predicted using prior knowledge about the sample in the form of a pre-trained deep neural network, thereby improving performance.

## Results

**Figure 2:**
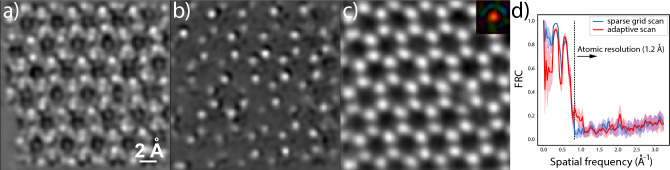
Ptychographic reconstruction of a MoS$_2$ data set with a scanning procedure that follows (**a**) a sparse grid scan, (**b**) an adaptive scan and (**c**) the conventional grid scan. While only 250 diffraction patterns are used in (**a**) and (**b**), the full data set with 10,000 diffraction patterns is used in (**c**). The inset in (**c**) illustrates the illumination probe used for the reconstruction with an estimated size of 0.93Å. The pixel size is identical to the one used in the reconstruction and its magnitude is represented by the intensity, and the phase is represented by the HSV colorwheel. (**d**) FRC of the sparse grid scan and the adaptive scan averaged over 25 data sets and where the standard deviation is illustrated by the shaded area.

The result of adaptive scanning on experimentally acquired MoS$_2$ data and its comparison to the result of a sparse grid scanning and the conventional grid scanning procedure is shown in Fig. 2. The data used for the comparison was not part of the training data for the adaptive scanning model. However, the entire data was acquired from the same sample and includes multiple data sets that were recorded from different regions of the sample. This data can be found in Ref.^28^. In our comparison, a ground truth reconstruction is obtained from one of the data sets each consisting of 10,000 diffraction patterns, while only 250 diffraction patterns have been used for the adaptive scanning as well as the sparse grid scanning reconstruction. Figure 2a shows the ptychographic reconstruction when using a sparse grid scanning procedure. The structure of the material is not clearly resolved and/or shows ambiguous features. Figure 2b shows the reconstruction when the scan positions are predicted through adaptive scanning. Although without the same homogeneous reconstruction quality throughout the entire field of view, the structure of the MoS$_2$ material is now much better resolved and is closer to the ground truth reconstruction of the full data grid scanning procedure, shown in Fig. 2c.

**Figure 3:**
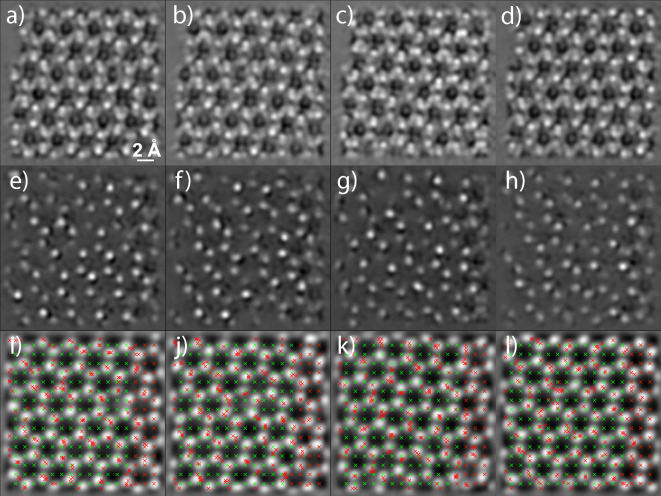
Ptychographic reconstructions of different MoS$_2$ data sets and with different scanning procedures. Reconstruction from 250 diffraction patterns of a data set that correspond to scan positions which follow (**a**–**d**) a sparse grid scanning sequence and (**e**–**h**) an adaptively predicted sequence. (**i**–**l**) Ground truth reconstruction of the full data set with 10,000 diffraction patterns shown with the scan positions used for the corresponding reconstructions (**a**–**d**) in green and (**e**–**h**) in red.

Further examples of reconstructions and their corresponding scan sequences are shown in Fig. 3. The results suggest that probe delocalization due to scattering plays an important role as to why an improved ptychographic reconstruction can be achieved by distributing the scan positions predominantly on the atoms of the specimen. This implies that similar results could be achieved by using RL with a reward function that specifically emphasizes the scattered electrons in the recorded diffraction patterns, which is an interesting area for future research.

The final point of our experimental investigation into adaptive scanning in ptychography evaluates the performance of the method for various prediction settings. We compare the Fourier ring correlation (FRC)^29^ as well as the structural similarity index measure (SSIM)^30^ between the reconstruction obtained from the reduced data and the ground truth reconstruction obtained from the full data to quantify the improvement of the effective image resolution and image quality when using adaptive scanning. In the first comparison, shown in Fig. 2d, we apply the FRC to the sparse grid scan and adaptive scan averaged over 25 data sets, respectively. For both cases, there is a sharp frequency cut off in the proximity of atomic resolution (1.2 Å). However, while the cross-correlation value almost disappears in the case of the sparse grid scan, it plateaus at a value of about 0.2 in the case of the adaptive scan. This result indicates the benefit in terms of achievable resolution of adaptive scanning in contrast to other low dose alternatives. In the latter comparison, SSIM$_a$ and SSIM$_s$ refer to reconstructions of reduced data obtained with the adaptive scanning and the sparse grid scanning procedure, respectively. Table 1 shows the relative reconstruction quality improvement $Q_{\text {SSIM}} = ( \text {SSIM}_a - \text {SSIM}_s ) / (\text {SSIM}_s)$ for different experimental settings averaged over the same 25 data sets as used before. In the case of 250 scan positions, which corresponds to a dose reduction by a factor of 40 with respect to the original data, tests were performed for different total numbers *T* of sub-sequences and therefore different amounts of scan positions included in each sub-sequence $\vec {{\textbf {R}}}_{P_t}$. The quality improvement ranges from $9.89\%$ to $15.84\%$ for 2 to 5 sub-sequences, respectively. Note that the scan positions of the first sub-sequence $\vec {{\textbf {R}}}_{P_0}$, provided to the RNN as part of the initial input, follow the sparse grid sequence and that the scan positions of each sub-sequence only cover a part of the sample, as can be seen in Fig. 6b. Further tests were performed using a larger number of total scan positions and 5 sub-sequences. However, the difference in quality between the reconstruction generated with the positions of the adaptive scan and the sparse grid scan decreases with the total number of positions used, as can be expected, since the sparse grid sampling covers the sampled area in an increasingly complete manner. These results indicate that the reconstruction quality improves with the frequency by which the positions are predicted, and that low dose experiments benefit the most from the adaptive scanning scheme.

**Table 1:** Performance of adaptive scanning for various experimental settings that differ in the number of scan positions and the total number of sub-sequences.

# Pos.	$N_\text {k}/N_\text {u}$	Dose ($\mathrm {e^-}$/Å$^{-2}$)	# Sub-seq.	Q$_{\text {SSIM}}$
250	8.21	$1.34 {{{\textsc {E}}}5 }$	2	$9.89 \pm 6.80 \%$
			3	$12.38 \pm 10.72 \%$
			4	$15.71 \pm 7.60 \%$
			5	$15.84 \pm 7.23 \%$
335	10.83	$1.79 {\textsc {e}}5$	5	$10.03 \pm 8.17 \%$
420	13.43	$2.25 {\textsc {e}}5$	5	$8.60 \pm 5.30 \%$
500	15.86	$2.68 {\textsc {e}}5$	5	$8.08 \pm 7.60 \%$

For each setting, the oversampling ratio $N_\text {k}/N_\text {u}$, which is calculated following Ref.^17^, and the electron dose is given.

**Figure 4:**
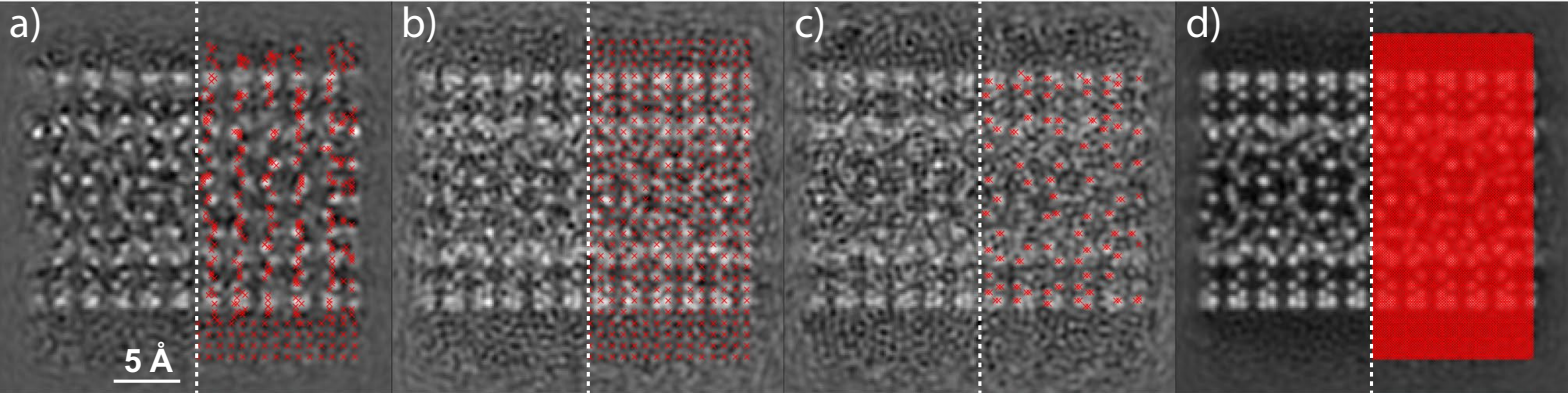
Ptychographic reconstruction of a DWCNT data set with a scanning procedure that follows (**a**) an adaptive scan, (**b**) a sparse grid scan, (**c**) an alternative low-dose scan and d) the conventional grid scan. 840 diffraction patterns have been used for the reconstructions in (**a**) and (**b**), 13,536 patterns were used for the reconstruction in (**c**) and the full data set consisting of 13,225 patterns was used for the reconstruction in (**d**). Half of the corresponding scan positions are illustrated on the right hand side of each reconstruction. Note that the alternative low-dose scan method used for generating (**c**) consists of a full grid scan at a very low dose and a consecutive scan using 311 scan positions that have been found to match the sample structure based on the result obtained from the previous scan. Only the latter scan positions are visualized in (**c**).

In Fig. 4, we compare the results of various scanning procedures using simulated double-walled carbon nanotube (DWCNT) data. This data is publicly available^28^. Figure 4a shows a ptychography reconstruction that considers 840 diffraction patterns that have been selected through the adaptive scanning procedure. Most of the unit cells of the structure can be resolved with a high contrast and therefore the configuration of the DWCNT can be easily deduced. The predicted scan positions coincide to a high degree with the structure of the DWCNT. Note that the initial scan sub-sequence visualized at the bottom of the reconstruction follows a sparse grid scan sequence. Figure 4b shows the reconstruction when using 840 diffraction patterns obtained from a sparse grid scanning procedure. The field of view is now much better covered with scan positions, but the periodicity at which the scan positions are spaced seems to be also present in the reconstruction. Hence, the reconstruction shows ambiguous features that make the interpretation of the structure more difficult compared to the previous case. Figure 4c shows the reconstruction of an alternative low-dose scanning procedure which has been described conceptually in Ref.^27^. Here, two consecutive scans are performed. The first scan is a conventional dense grid scan consisting of 13,225 diffraction patterns with an electron dose of $4{\textsc {e}}3$ $\mathrm {e^-}$/Å$^{-2}$ compared to $1{\textsc {e}}5$ $\mathrm {e^-}$/Å$^{-2}$. The same scan with the latter dose has also been used for the dense grid scan in Fig. 4d. The second scan of the alternative low-dose scanning procedure was limited to 311 scan positions as to match the total electron dose of the procedures in Fig. 4a and b. An atom finding method was applied to the ptychography reconstruction generated after the first scan to adapt these 311 scan positions to the atomic structure of the DWCNT specimen. Although the predicted scan positions in this approach match the atomic structure almost perfectly, their contribution to the final reconstruction seems to be not sufficient given that most of the total electron dose is required for their optimal prediction. Quantitatively, we obtain a relative reconstruction quality improvement $Q_{\text {SSIM}}$ of $2.60 \pm 3.38\%$ and $16.97 \pm 3.49\%$ when using adaptive scanning with respect to the sparse grid scanning and alternative low-dose scanning procedure, respectively.

## Conclusion

We have presented a method for electron ptychography that reduces the electron dose through adaptive scanning. Sub-sequences of scan positions are predicted by the model within milliseconds, allowing an acquisition rate that theoretically exceeds rates currently achieved in 4D-STEM experiments. The method would therefore have the potential to be applied in real time at the microscope if the used ptychography reconstruction algorithm could generate images sufficiently fast. Future work does therefore require either an improvement of iterative ptychography algorithms in terms of processing speed or the integration of direct ptychography reconstruction methods, such as single-sideband (SSB) ptychography^31^, in the adaptive scanning workflow. We show an improved resolution and reconstruction quality when using an adaptive scanning approach on experimentally acquired monolayer MoS$_2$ data sets in comparison to another dose reduction scanning approach. These improvements show that adaptive scanning for ptychography is a useful technique to lower the dose needed for the analysis of sensitive samples. It can be provided with simulated or generic experimental training data^32,33^ to increase its applicability to a variety of different or less periodic material structures. We have demonstrated the generalizability of our method by applying it to simulated DWCNT data sets and showing that it outperforms other low-dose alternatives. In addition, the proposed workflow can be taken as a blueprint for a broad range of scanning microscopy methods and thus paves the way for future research in machine learning supported, automated and autonomous microscopy^34,35^.

## Methods

### Image formation in ptychography

The approach described in this paper is compatible with multisclice ptychography, but in light of the application to a 2D material we constrain ourselves to single-slice ptychography. Here, ptychography can be expressed by a multiplicative approximation that describes the interaction of a wavefunction $\psi ^{in}_p({\textbf {r}})$ of an incoming beam with the transmission function $t({\textbf {r}})$ of a specimen. For each measurement *p*, the beam is shifted by ${\textbf {R}}_p$ and a diffraction pattern is acquired with the intensity $I_p$ that is expressed by

1 $$\begin{aligned} \begin{aligned} I_p = |\Psi ^{ex}_p({\textbf {k}})|^2 = |\mathscr {F} \left[ \psi ^{in}_p({\textbf {r}}-{\textbf {R}}_p) t({\textbf {r}}) \right] |^2, \end{aligned} \end{aligned}$$

where $\mathscr {F}$ is the Fourier propagator, ${\textbf {r}}$ the real space coordinate, ${\textbf {k}}$ the reciprocal space coordinate and $\Psi ^{ex}_p({\textbf {k}})$ the exit wavefunction at the detector. The transmission function can be defined as $t({\textbf {r}}) = e^{i \sigma V({\textbf {r}})}$, with the interaction constant $\sigma$ and the complex potential $V({\textbf {r}})$. Throughout this treatment, $\sigma$ is absorbed into $V({\textbf {r}})$. X-ray and optical ptychography is mathematically described similarly with the only difference that the transmission function $t({\textbf {r}})$ is related to the complex refractive index of the specimen. Figure 1a illustrates the experimental configuration of conventional ptychography. The potential of the specimen is recovered from data of experimentally acquired diffraction patterns $J_p$ using a reconstruction algorithm. Here, we apply a gradient based algorithm^17^ with a gradient decent optimization and the potential is retrieved by iteratively minimizing the loss function

2 $$\begin{aligned} \mathscr {L}(V) = \frac{1}{P}\sum ^P_{p=1} \Vert I_p(V) - J_p\Vert ^2_2 . \end{aligned}$$

### Generation of scan sequences

We use a recurrent neural network (RNN)^36–38^ for the generation of scan sequences. Its network architecture is designed to model temporal sequences with recurring input information. Memory cells combine the current input information $X_t$ with the hidden state $H_t$ and map it to the next hidden state $H_{t+1}$. These hidden states represent the memory gathered from all the previous time steps. Gated recurrent units (GRU)s^39^, which allow a computationally fast mapping with a high performance, are used in this work. At every time step *t*, an output is generated on the basis of the current hidden state. In the implementation shown here, the output corresponds to multiple scan positions, i.e. a sub-sequence of scan positions, given by a vector of 2D coordinates $\vec {{\textbf {R}}}_{P_t}$. In principle, the output can be reduced to a single scan position ${\textbf {R}}_{p_t}$, but we do not do so for practical reasons that involve a reduced training performance of the network and also a greatly increased acquisition time due to, e.g., more frequent data transfer and pre-processing of these intermediate data chunks. The sub-sequence is predicted via a fully connected layer (FC) that is parameterized by the layer weights $\theta _H$:

3 $$\begin{aligned} \vec {{\textbf {R}}}_{P_t} = \text {FC}_{\theta _H}(H_t). \end{aligned}$$

At the predicted scan positions $\vec {{\textbf {R}}}_{P_t}$, diffraction patterns ${\varvec{J}}_{P_t}$ are acquired by the microscope and from these diffraction patterns a potential $V_t({\textbf {r}})$ is reconstructed minimizing Eq. (2). The intermediate reconstruction $V_t({\textbf {r}})$ combined with its corresponding sub-sequence of scan positions $\vec {{\textbf {R}}}_{P_t}$ can then be used for the input information $X_t$ of the RNN. However, the bandwidth of the information given in $V_t({\textbf {r}})$ and $\vec {{\textbf {R}}}_{P_t}$ differs strongly and thus pre-processing is required before the two components can be concatenated and mapped to $X_t$. For the processed location information ${\textbf {L}}_t$ based on the sub-sequence $\vec {{\textbf {R}}}_{P_t}$, a FC that is parameterized by the weights $\theta _R$ is used:

4 $$\begin{aligned} {\textbf {L}}_t = \text {FC}_{\theta _R}(\vec {{\textbf {R}}}_{P_t}). \end{aligned}$$

For the processed structure information ${\textbf {C}}_t$ based on the reconstructed potential $V_t({\textbf {r}})$, a compressed representation $z_t$ is generated by using the encoder part of a convolutional autoencoder^40^. This processing step is shown in Fig. 1b and the training of the convolutional autoencoder is described in the Supplementary Information. The compressed representation $z_t$ is then fed into a FC that is parameterized by the weights $\theta _z$:

5 $$\begin{aligned} {\textbf {C}}_t = \text {FC}_{\theta _z}(z_t). \end{aligned}$$

The processed location information ${\textbf {L}}_t$ is subsequently concatenated with the processed structure information ${\textbf {C}}_t$ and mapped to the input information $X_t$ with a FC that is parameterized by the weights $\theta _{LC}$. The whole process of predicting sub-sequences of scan positions and acquiring the corresponding diffraction patterns is repeated until a ptychographic data set of desired size is reached. Finally, backpropagation through time (BPTT) is used to generate the required gradients to update the network weights $\theta = \{ \theta _H,\theta _{\text {GRU}},\theta _{LC},\theta _R, \theta _z \}$ of the RNN. Figure 1c shows the prediction process modeled by the RNN in full detail.

### Training through reinforcement learning

A RNN can be combined with RL to provide a formalism for modelling behaviour to solve decision making problems. In the case of adaptive scanning in ptychography, where we seek to predict multiple scan positions at each time step, RL can be formalized by a partially observable stochastic game (POSG) that is described by a 8-tuple, $\langle M, \mathscr {S},\{\mathscr {A}^m\}_{m\in M},\rho ,\{r^m\}_{m\in M}, \{\mathscr {O}^m\}_{m\in M}, \omega , \gamma \rangle$, with multiple agents *M*. At each time-step *t* an agent *m* selects an action $a^m_t \in \mathscr {A}^m$ and makes an observation $o^m_t \in \mathscr {O}^m$ given the state $s_t\in \mathscr {S}$. Thus, joint actions ${\varvec{a}}_t = \langle a^1_t,\ldots , a^m_t \rangle$ from the joint action space ${\varvec{\mathscr {A}}} = \mathscr {A}_1 \times \cdots \times \mathscr {A}_M$ are executed and joint observations ${\varvec{o}}_t = \langle o^1_t, \ldots , o^m_t \rangle$ from the joint observation space ${\varvec{\mathscr {O}}} = \mathscr {O}_1 \times \cdots \times \mathscr {O}_M$ are received from the environment at every time step. The next state $s_{t+1}$ is generated according to a transition function $\rho : \mathscr {S} \times {\varvec{\mathscr {A}}} \times \mathscr {S} \rightarrow [0,1]$, the observations ${\varvec{o}}_{t+1}$, containing incomplete information about the state $s_{t+1}$, are generated according to an observation function $\omega : {\varvec{\mathscr {A}}} \times \mathscr {S} \times {\varvec{\mathscr {O}}} \rightarrow [0,1]$ and each agent receives its immediate reward defined by the reward function $r^m: \mathscr {S} \times {\varvec{\mathscr {A}}} \rightarrow \mathbb {R}$. This reward $r^m$ contributes to the total reward computed at the end of the sequence, $G^m=\sum ^T_{t=0} \gamma ^{t} r^m({\varvec{a}}_t,s_t)$, also known as the return. The discount factor $\gamma \in [0,1]$ controls the emphasis of long-term rewards versus short-term rewards. The entire history of observations and actions up to the current time ${\varvec{h}}_t= \{ {\varvec{o}}_1, {\varvec{a}}_1, \ldots , {\varvec{o}}_{t-1},{\varvec{a}}_{t-1},{\varvec{o}}_t \}$ is used as basis for optimal or near-optimal decision making. A stochastic policy $\pi _{\theta ^m}(a^m_t|{\varvec{h}}_t)$ maps the history of past interactions ${\varvec{h}}_t$ to action probabilities. Given a continuous action space, the policy can be represented by a two-dimensional Gaussian probability distribution:

6 $$\begin{aligned} \pi _{\theta ^m}(a^m_t|{\varvec{h}}_t) = \mathscr {N}({\varvec{\mu }}_{\theta ^m}({\varvec{h}}_t), \Sigma ), \end{aligned}$$

with its mean vector ${\varvec{\mu }}_{\theta ^m}({\varvec{h}}_t)$ corresponding to ${\textbf {R}}_{p_t}$, where the history ${\varvec{h}}_t$ is summarized in the hidden state $H_t$ of the RNN and the covariance matrix $\Sigma$ with fixed variances $\sigma _x^2 \in [0,1]$ and $\sigma _y^2 \in [0,1]$. The joint policy of all agents is then defined as ${\varvec{\pi }}_{{\varvec{\theta }}}({\varvec{a}}_t| {\varvec{h}}_t) = \prod _{m=1}^{M} \pi _{\theta ^m}(a^m_t|{\varvec{h}}_t)$, with ${\varvec{\theta }}=\{ \theta ^m \}_{m \in M}$. The goal of RL is now to learn a joint policy that maximizes the expected total reward for each agent *m* with respect to its parameters $\theta ^m$:

7 $$\begin{aligned} \begin{aligned} \mathscr {J}^m({\varvec{\theta }}) = \mathbb {E}_{{\varvec{\pi }}_{{\varvec{\theta }}}({\varvec{\tau }})} \left[ G^m \right] \approx \frac{1}{N} \sum ^N_{n=1} G^m_n, \end{aligned} \end{aligned}$$

where the expected total reward can be approximated by Monte Carlo sampling with *N* samples. In this paper, improvement of the policy is achieved by updating the policy parameters $\theta ^m = \{ \theta ^m_H,\theta _{\text {GRU}},\theta _{LC},\theta _R, \theta _z \}$ with ’REINFORCE’^41^, a policy gradient method:

8 $$\begin{aligned} \nabla _{\theta ^m} \mathscr {J}^m({\varvec{\theta }})= & {} \mathbb {E}_{{\varvec{\pi }}_{{\varvec{\theta }}}({\varvec{\tau }})} \left[ \nabla _{\theta ^m} \text {log} {\varvec{\pi }}_{{\varvec{\theta }}}({\varvec{\tau }}) G^m \right] \nonumber \\{} & {} \approx \frac{1}{N} \sum ^N_{n=1} \sum ^T_{t=0} \nabla _{\theta ^m} \text {log} \pi _{\theta ^m}(a^m_{n,t}|{\varvec{h}}_{n,t}) G^m_n. \end{aligned}$$

The derivation of $\nabla _{\theta ^m} \mathscr {J}^m({\varvec{\theta }})$ is given in the Supplementary Information.

### Learning to adaptively scan in ptychography

While policy gradient methods are the preferred choice to solve reinforcement learning problems in which the action spaces are continuous^42^, they come with significant problems. Like any gradient based method, policy gradient solutions mainly converge to local, not global, optima^43^. In this paper, we reduce the effect of this problem during training by splitting the training of the RNN into supervised learning and RL. While training in RL can be performed with a policy whose parameters are arbitrarily initialized, this is not ideal. Having an adequate initial guess of the policy and using RL subsequently to only fine-tune the policy is a much easier problem to solve. A sparse grid scan sequence is a reasonable initialization as it follows the current scanning convention used in a microscope. Pre-training of the parameterized policy for the RL model can then be performed by supervised learning applied on the RNN such that the discrepancy between the predicted scan positions $\vec {{\textbf {R}}}_{P_t} = \vec {{\varvec{\mu }}}_{{\varvec{\theta }}}({\varvec{h}}_t)$ and the scan positions of the initialization sequence $\vec {{\textbf {R}}}^{\text {init}}_{P_t}$ is minimized:

9 $$\begin{aligned} \mathscr {K}({\varvec{\theta }}) = \sum ^T_t \Vert \vec {{\textbf {R}}}_{P_t} - \vec {{\textbf {R}}}^{\text {init}}_{P_t} \Vert ^2_2. \end{aligned}$$

**Figure 5:**
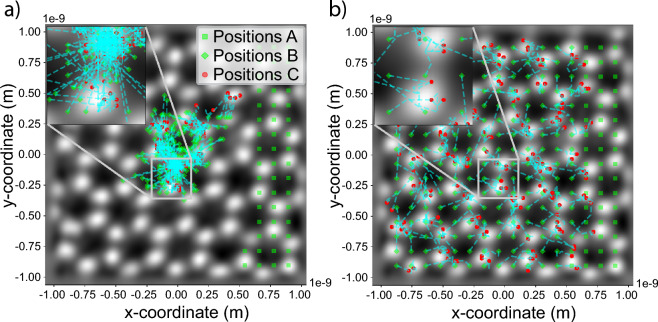
Fine-tuning of a policy with RL that (**a**) has not been initialized and (**b**) has been initialized via supervised learning. In the latter case, the training starts with a sequence that matches a sparse grid scan sequence. Positions A indicate the scan positions of the first sub-sequence $\vec {{\textbf {R}}}_{P_0}$ that is provided to the RNN as part of the initial input. Positions B and C are the scan positions of all predicted sub-sequences at iteration 0 and 10,000, respectively. The trajectories they form during the optimization process are indicated by dashed blue lines.

Figure 5 illustrates the scan positions during the fine-tuning of the policy through RL for the first 10,000 iterations when either (a) a policy that has not been initialized via supervised learning or (b) an initialized policy is used. While the scan positions in both cases converge to the atomic structure, the positions predicted by the non-initialized policy are distributed only within a small region of the field of view during the entire training. Note that once the predicted scan sequence mimics the sparse grid scan sequence as a result of the supervised learning based initialization, all further improvements in performance are the result of the subsequent training through RL.

**Figure 6:**
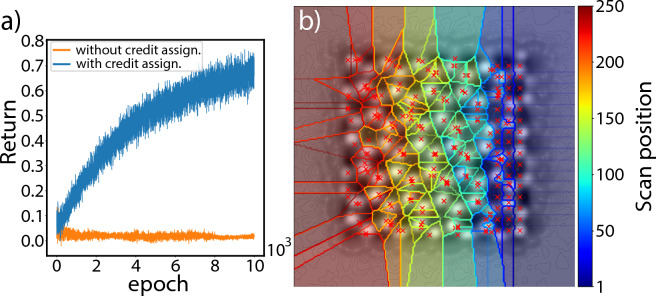
(**a**) Learning curves for the first 10,000 iterations of RL with multiple agents and without credit assignment or with credit assignment, illustrated in orange and blue, respectively. (**b**) A Voronoi diagram is used to assign a unique reward to each scan position of the predicted sequence. The scan positions are shown as red dots, where the first 50 positions are distributed on the right side within the dark blue area. For visualization purpose, the ground truth reconstruction is included in the diagram.

A high variance of gradient estimates is another problem that particularly strongly affects the Monte Carlo policy gradient method^42,44,45^. Due to this, the sampling efficiency is relatively low, which causes a slow convergence to a solution. This makes deep RL applied to ptychography challenging as the image reconstruction itself requires iterative processing. The high variance can be in part attributed to the difficulty of assigning credit from the overall performance to an individual agent’s action. Here, we introduce a way to estimate the reward function in order to tackle the credit assignment problem for adaptive scanning in ptychography. The reward function should naturally correspond to the quality of the ptychographic reconstruction. We have found empirically that a high reconstruction quality correlates positively with a high dynamic range in the phase. Therefore, the reward function could intuitively be formalized by $r^m({\varvec{a}}_t|s_t)=P^{-1} \sum _{{\textbf {r}}\in \text {FOV}} V({\textbf {r}})$, where *P* is the total number of scan positions. This formulation, however, does not solve the credit assignment problem and results in an insufficient training performance, as shown in Fig. 6a. To estimate the reward for the actions of each individual agent, we use a tessellation method that partitions the atomic potential into small segments. A Voronoi diagram^46^, where each position corresponds to a seed for one Voronoi cell, enables assignment of only a part of the total phase to each position. More precisely, the Voronoi diagram formed by the predicted scan positions is overlaid with the corresponding ptychographic reconstruction at the end of the prediction process and the summed phase within each Voronoi cell is the reward for that cell’s seed position. The reward function can be expressed by $r^m({\varvec{a}}_t|s_t)= P^{-1} \sum _{{\textbf {r}}\in \text {Cell}^m} V({\textbf {r}})$. Figure 6b shows a Voronoi diagram generated by predicted scan positions.

### Settings

For the experimental investigation, we acquired multiple ptychographic data sets from a monolayer molybdenum disulfide (MoS$_2$) specimen with a Nion HERMES microscope. The microscope was operated with a 60 kV acceleration voltage, a convergence angle of 33 mrad and diffraction patterns with a pixel size of 0.84 mrad were acquired using a Dectris ELA direct-electron detector mounted at the electron energy-loss spectroscopy (EELS) camera port. Distortions induced by the EEL spectrometer were corrected with in-house developed software. For the ptychographic data set acquisition, a conventional grid scan with a scanning step size of 0.02 nm was used. From the experimentally acquired data sets we created 200 smaller data sets, each with 10,000 diffraction patterns and located at different regions of the sample. 175 of these small data sets were dedicated to the training of the network model, while the remaining 25 data sets were used to test the trained model and to generate the results shown here. The diffraction patterns were binned by a factor of 2 to $64 \times 64$ pixels. The adaptive scanning model was trained on the small data sets with the goal of predicting optimal scan sequences of 250 to 500 probe positions, out of the possible 10,000, which corresponds to a dose reduction by a factor of 40 to 20. Each sub-sequence contains 50 to 100 positions, where the initially given first sub-sequence follows a sparse grid scanning sequence.

We conducted a second investigation of the model’s performance on simulated data sets of a DWCNT and compared it to the performance of alternative low-dose data acquisition methods in ptychography. The data sets were generated using the simulation tool *ab*TEM^47^ where we set the acceleration voltage to 60 kV, the convergence angle to 40 mrad and the scanning step size to 0.02 nm. The size of the diffraction patterns is $86 \times 86$ pixels with a pixel size of 0.91 mrad. 962 data sets have been used to train the network model and from the 13,225 diffraction patterns in each data set only 840 were chosen within the prediction process of the adaptive scanning workflow. 25 data sets were used to compare the different scanning procedures and analyse their performance using the $Q_{\text {SSIM}}$ metric. The simulated DWCNT consisted of an inner and an outer nanotube with a diameter of 9.78 Å and 16.44 Å and a chiral angle of $44^{\circ }$ and $60^{\circ }$, respectively. For each data set, a unique rotation between the two nanotubes and a translation of the entire DWCNT within the field of view was applied.

The ptychographic reconstructions were performed with an optimized version of ROP^17,28^ that allows simultaneous reconstruction from a batch of different data sets and therefore the parallel hardware architecture of a NVIDIA V100 GPU could be optimally used to efficiently train the model. For a batch size of 24, reconstructions were retrieved in about 35 s. A gradient descent step size $\alpha _{\text {ROP}}$ of 525 was chosen and the potential was retrieved at iteration 5. In the experimental investigation, the reconstructed potential was $200\times 200$ pixels with a pixel size of 0.0154 nm, for a field of view of $2 \times 2$ nm, while for the simulation, the reconstructed potential was $200\times 200$ pixels with a pixel size of 0.0140 nm, for a field of view of $2.3 \times 2.3$ nm. For the generation of the reward function, Voronoi diagrams were generated with the Jump Flooding Algorithm^48^ and for the implementation of the network models, PyTorch^49^ was used. For the compression of structure information, we used a convolutional autoencoder consisting of 6 convolutional layers with kernels of dimension 3, a stride of 1 and channels that ranged from 16 to 512 for the encoder and decoder part, respectively. The input of the autoencoder had a dimension of 512 with a pixel size of 0.0064 nm and thus a scaling and an interpolation was required before the potential generated by ROP could be compressed. In addition, the value of the potential $V_i$ at each pixel *i* was transformed to zero mean and unit variance. For the prediction of the scan sequences, pre-training and fine-tuning was performed with a RNN model composed of 2 stacked GRU layers with hidden states $H_t$ of size 2048, the Adam optimizer^50^ with a learning rate $\alpha _{\text {RNN}}$ of $1{{\textsc {e}-}}5$ and a batch size of 24. For the fine-tuning, a policy with variances of $\sigma _x^2 = \sigma _y^2 = 0.0125^2$ was chosen and a myopic behavior was enforced by setting the discount factor for the return, *G*, to $\gamma = 0$. In the case of the experimental investigation, training of the autoencoder involved 100,000 iterations, while the training of the RNN with supervised learning and RL has been performed with 800 and 20,000 iterations, respectively. In the second investigation with the simulated data, the training of the autoencoder has been done with 30,000 iterations and for the training of the RNN with supervised learning and RL, 200 and 2800 iterations were used, respectively.

## Supplementary Information

Supplementary Information.

## Data Availability

The source code of the presented work is available at Gitlab^51^ and the experimental and simulated data can be found in Ref.^28^. All other code is available from the corresponding author on reasonable request.
