# Supplementary material for: Deep reinforcement learning for data-driven adaptive scanning in ptychography
Source: Sci Rep. 2023 May 30;13:8732. doi: 10.1038/s41598-023-35740-1 (PMC10229550; doi:10.1038/s41598-023-35740-1)
Supplement: Supplementary file 1 — Supplementary Information. [file 41598_2023_35740_MOESM1_ESM.pdf]

Supplementary Information:
Deep Reinforcement Learning for Data-Driven Adaptive Scanning in Ptychography

Marcel Schloz,[1] Johannes Müller,[1] Thomas C. Pekin,[1] Wouter Van den Broek,[1] Jacob Madsen,[2] Toma Susi,[2] and Christoph T. Koch[1]

[1] *Humboldt Universität zu Berlin, Institute of Physics & IRIS Adlershof, Newtonstraße 15, 12489 Berlin, Germany*
[2] *University of Vienna, Faculty of Physics, Boltzmanngasse 5, 1090 Vienna, Austria*

S1. STRUCTURE CONTENT COMPRESSION THROUGH CONVOLUTIONAL AUTOENCODERS

For the processing of the intermediate reconstruction $V_t(\mathbf{r})$ that is the basis for the compressed representation z_t of the RNN, we make use of a convolutional autoencoder [1], which is a deep learning model based on convolutional neural networks [2]. This model is composed of two parts, namely an encoder and a decoder. The encoder transforms potentially degraded input data into a compressed representation and the decoder recovers the original input from this representation [3]. By reducing the dimensionality, the model ensures that only the most important information for the reconstruction is extracted. In the convolutional autoencoder applied here, an image $V \in \mathbb{R}^{H\times W\times 2}$ reconstructed from diffraction patterns is mapped to the latent representation $z \in \mathbb{R}^{\frac{H}{2^b}\times\frac{W}{2^b}\times f}$ by using the encoder network E_{ϕ_e}:

$$z = E_{\phi_e}(V), \tag{S.1}$$

where ϕ_e corresponds to the encoder network weights. The encoder network consists of a concatenation of b convolution layers that increase the feature space f and halve the dimension size $H \times W$. The latent representation z is then mapped back to the original dimensions by the decoder network

$$\hat{V} = D_{\phi_d}(z), \tag{S.2}$$

where ϕ_d are the decoder network weights and $\hat{V}$ is the predicted image. Transposed convolutional layers form the decoder network, which has the inverse effect to the layers used in the encoder network. Figure S1 illustrates the encoder-decoder architecture in full detail.

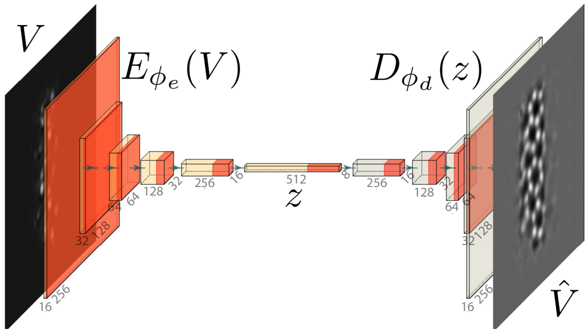

FIG. S1. Schematic of the convolutional autoencoder model. A reconstruction V generated from diffraction patterns is mapped to the compressed representation z by using the encoder network $E_{\phi_e}(V)$. The compressed representation z is then basis for a reverse mapping by the decoder network $D_{\phi_d}(z)$ to generate a prediction of the potential $\hat{V}$.

After estimating the network weights ϕ_e and ϕ_d by minimizing the loss function

$$\mathcal{M}(\phi_e, \phi_d) = \|D_{\phi_d}(E_{\phi_e}(V)) - V\|_2^2, \tag{S.3}$$

we can utilize the encoder network E_{ϕ_e} for the compression of $V_t(\mathbf{r})$. Figure S2 shows a compression of a partial reconstruction $V_t(\mathbf{r})$ and the decompression of its corresponding compressed representation z_t. This preprocessing helps the algorithm form the hybrid input information X_t by reducing the structure input information size, but also handle reconstructions from experimentally acquired data that may suffer from noise, contamination and/or incorrect scan positions.

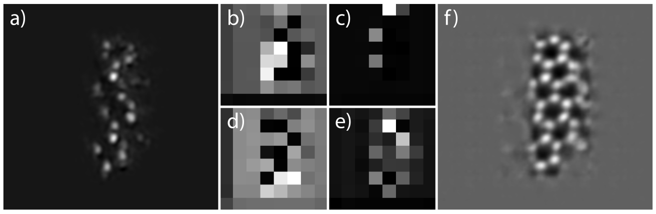

FIG. S2. Convolutional autoencoder applied on partial structure information, given by the reconstruction of data from a sub-sequence of scan positions. a) The reconstruction $V_t(\mathbf{r})$ from a sub-sequence of scan positions that is used as input for the convolutional autoencoder. b-e) 4 channels of the compressed representation z_t of the structure information. f) Decoded structure information $\hat{V}_t$ from z_t.

S2. THE "REINFORCE" ALGORITHM

In the case of the multi-agent RL problem, where we use the POSG formalism, the objective of an agent m given by Eq. (7) can be expressed by

$$\mathcal{J}^m(\boldsymbol{\theta}) = \mathbb{E}_{\boldsymbol{\pi_\theta}(\boldsymbol{\tau})}\left[G^m\right] = \int \boldsymbol{\pi_\theta}(\boldsymbol{\tau}) G^m d\boldsymbol{\tau}, \qquad \text{(S.4)}$$

with the trajectory $\boldsymbol{\tau} = \{s_0, \boldsymbol{o}_0, \boldsymbol{a}_0, s_1, ..., s_T, \boldsymbol{o}_T, \boldsymbol{a}_T\}$ and the policy induced trajectory distribution $\boldsymbol{\pi_\theta}(\boldsymbol{\tau}) = q(s_0) \prod_{t=0}^{T} \rho(s_{t+1}|s_t, \boldsymbol{a}_t) \boldsymbol{\pi_\theta}(\boldsymbol{a}_t|\boldsymbol{h}_t) \omega(\boldsymbol{o}_t|s_t)$ and where $q(s_0)$ is the distribution of initial states. Applying the gradient ∇_{θ^m} to the objective and using the identity $\nabla_{\boldsymbol{\theta}} \boldsymbol{\pi_\theta}(\boldsymbol{\tau}) = \boldsymbol{\pi_\theta}(\boldsymbol{\tau}) \nabla_{\boldsymbol{\theta}} \log \boldsymbol{\pi_\theta}(\boldsymbol{\tau})$, we obtain:

$$\begin{aligned} \nabla_{\theta^m} \mathcal{J}^m(\boldsymbol{\theta}) &= \int \boldsymbol{\pi_\theta}(\boldsymbol{\tau}) \nabla_{\theta^m} \log \boldsymbol{\pi_\theta}(\boldsymbol{\tau}) G^m d\boldsymbol{\tau} \\ &= \int \boldsymbol{\pi_\theta}(\boldsymbol{\tau}) \nabla_{\theta^m} \log [q(s_0) \prod_{t=0}^{T} \rho(s_{t+1}|s_t, \boldsymbol{a}_t) \\ &\quad \times \prod_{m=1}^{M} \pi_{\theta^m}(a_t^m|\boldsymbol{h}_t) \omega(\boldsymbol{o}_t|s_t)] G^m d\boldsymbol{\tau} \\ &= \mathbb{E}_{\boldsymbol{\pi}_\theta(\boldsymbol{\tau})} \left[\sum_{t=0}^{T} \nabla_{\theta^m} \log \pi_{\theta^m}(a_t^m|\boldsymbol{h}_t) \right. \\ &\quad \left. \times \left(\sum_{t'=t}^{T} \gamma^{t'-t} r^m(\boldsymbol{a}_{t'}, s_{t'}) \right) \right]. \end{aligned} \qquad \text{(S.5)}$$

S3. SETTINGS

All settings used for training the adaptive scanning model are summarized in Table S1.

TABLE S1. Settings for the potential reconstruction with ROP, structure content compression with the convolutional autoencoder and prediction of the scan sequences with the RNN.

ROP	MoS_2 / DWCNT
acceleration voltage (kV)	60
convergence angle (mrad)	33 / 40
object dimension (px)	200
real space pixel size (nm)	0.0154 / 0.0140
diffraction pattern dimension (px)	64 / 86
reciprocal space pixel size $\left(\text{nm}^{-1}\right)$	0.4759 / 0.3913
scanning step size (nm)	0.02
iterations	5
step size α_{ROP}	5.25E2
batch size	24
**Conv. autoencoder**	**MoS_2 / DWCNT**
input dimension (px)	512
pixel size (nm)	0.0064
encoder/decoder kernel sizes (px)	$[3, 3, 3, 3, 3, 3]$
encoder/decoder kernel strides (px)	$[1, 1, 1, 1, 1, 1]$
encoder output channels	$[16, 32, 64, 128, 256, 512]$
decoder output channels	$[256, 128, 64, 32, 16, 2]$
iterations	100000 / 30000
step size α_{CAE}	1E-5
batch size	24
**RNN**	**MoS_2 / DWCNT**
sequence length	250 / 840
sub-sequence length	50 / 105
hidden state H_t size	2048
stacked GRU layers	2
iterations (superv. L. + RL)	800 + 20000 / 200 + 2800
step size α_{RNN}	1E-6
batch size	24

ADDITIONAL INFORMATION

All correspondence should be addressed to schlozma@hu-berlin.de.

REFERENCES

[1] Jonathan Masci et al. "Stacked convolutional auto-encoders for hierarchical feature extraction". In: *International conference on artificial neural networks.* Springer. 2011, pp. 52–59.

[2] Yann LeCun et al. "Backpropagation applied to handwritten zip code recognition". In: *Neural computation* 1.4 (1989), pp. 541–551.

[3] Geoffrey E Hinton and Ruslan R Salakhutdinov. "Reducing the dimensionality of data with neural networks". In: *science* 313.5786 (2006), pp. 504–507.
